# Stokes-Adams Syndrome Produced by Digitalis in a Case of Auricular Fibrillation

**Published:** 1930

**Authors:** T. F. R. Hewer

**Affiliations:** Late House Physician, Radcliffe Infirmary, Oxford


### STOKES-ADAMS SYNDROME PRODUCED BY DIGITALIS IN A CASE OF AURICULAR FIBRILLATION.

BY

T. F. R. Hewer, M.D.,

*Late House Physician, Radcliffe Infirmary, Oxford.*

---

A number of cases have been reported from time to time in which heart-block has been produced by digitalis therapy, but I have been unable to discover any reference to the occurrence of the Stokes-Adams syndrome under these conditions. The following case may, for that reason, be of interest :—

The patient was a married woman, aged 37, who had a definite history of rheumatic fever at the age of 23. She was in good health, had two children, and suffered from no cardiac symptoms till eighteen months ago, when she began to have dyspnœa on slight exertion, this became progressively worse till she was confined to bed eleven months ago with orthopnœa. Nine months ago œdema of the legs and ascites appeared and persisted, although she was still in bed. There were occasional attacks characterized by a feeling of tightness in the chest, followed by profuse sweating and retching, which lasted a few seconds and were not accompanied by any loss of consciousness. Such attacks occurred at almost weekly intervals, irrespective of the treatment she was receiving ; there was no evidence that they were due to digitalis, as this drug was found to have very little beneficial effect, and was only exhibited at intervals in small doses. For the two months prior to admission to hospital she was taking one Nativelle granule daily, and there had been no attacks for the past three weeks. Unfortunately, there is no definite record as to the date of onset of auricular fibrillation, but apparently it had been present for quite twelve months.

On 6th March, 1930, the patient was admitted in a state of exhaustion, with orthopnœa, considerable ascites, and

œdema of the lower extremities. She was fibrillating and the pulse was hardly perceptible, with a rate about 85. The heart dullness extended from the mid-line to the anterior axillary line. A loud systolic bruit was heard at the apex and at the aortic base. Blood pressure, 105/95.

In view of the increasing ascites and her exhausted condition it was thought advisable to try the effect of digitalisation. She was given thirty minims of the tincture four-hourly for six doses, and showed considerable improvement; the dose was then decreased to twenty minims thrice daily for three doses, on the third day, and her condition was then very satisfactory; there was no nausea and the pulse was more steady at a rate of 72, but still fibrillating. By this time she had, therefore, been given three drachms of the tincture in forty-eight hours.

During the night following the last dose of digitalis the pulse suddenly became regular at 36, and there were six Stokes-Adams attacks within the course of one and a half hours. Each of these began with a feeling of faintness for a few seconds, followed by vomiting and then complete unconsciousness with generalized convulsions for 15–20 seconds, during which time there was no ventricular systole. There was complete recovery between the attacks. Adrenalin was given with success, but the pulse remained regular at 36 for several hours. No more digitalis was given.

On the fourth day (that following these attacks) the general condition was fairly good; the pulse was 70, with occasional dropped beats but no auricular fibrillation.

On the evening of the fifth day the patient became much worse and had *pulsus alternans* for four hours. Unfortunately no pulse tracing was taken, but the beats felt equally spaced, and it did not appear to be "coupling."

On the sixth day there were occasional dropped beats, and on the seventh auricular fibrillation reappeared. Coincidentally with the return of fibrillation the patient's condition improved, dyspnœa was less and there was a slight diminution of ascites, with some increase of urine excretion.

There was then no change till the twenty-third day, when extreme cyanosis and dyspnœa developed and the patient died. Permission was not given for a post-mortem.

I am indebted to Dr. A. G. Gibson for his kindness in allowing me to report this case.

